# Implicit intervention approach for empathy: exploring the combined effects of empathic concern and visual perspective-taking

**DOI:** 10.3389/fpsyt.2025.1530532

**Published:** 2025-05-30

**Authors:** Taesun Kim, HeungSik Yoon, Sang Hee Kim

**Affiliations:** Affective Cognition Laboratory, Department of Brain and Cognitive Engineering, Korea University, Seoul, Republic of Korea

**Keywords:** empathy, empathic concern, visual perspective-taking, implicit intervention, attentional disengagement, helping intentions

## Abstract

Interest in fostering empathy has expanded rapidly recently, with increasing recognition of its role in emotional wellbeing and positive social outcomes. The current study investigated whether interventions to enhance empathic concern and visual perspective taking would influence socioemotional information processing and enhance empathic responses toward others. For this purpose, we devised two implicit intervention tasks: the gamified implicit compassion promotion task (gam-iCPT) to target empathic concern, and the other-oriented visual perspective-taking task (OVPT) to target visual perspective taking. A total of 128 healthy adults were randomly assigned to one of four intervention groups: the combined, single gam-iCPT, single OVPT and control group. Intervention outcomes were assessed using the dot-probe attention bias task with facial expressions of emotions and the empathy rating task featuring distressed others. We found consistent differences in outcome measures between the combined intervention group and the single gam-iCPT group. Specifically, the combined group showed faster disengagement from emotional faces, as well as greater empathic concern and increased helping intentions toward sad victims, compared to the single gam-iCPT group. However, no significant intervention effects were observed when compared to the control group. These results suggest that implicit interventions to target both empathic concern and visual perspective taking together may have a potential impact on empathy-related socioemotional processing compared to targeting each single element. The absence of significant effects relative to the control group, however, highlight the complexity of mechanisms underlying empathy enhancement, warranting further investigations.

## 1 Introduction

Empathy is a well-known predictor of prosocial behavior, social relation, and overall life satisfaction (1–3). Empathy is multifaceted construct that comprises several distinct but related components (4–10). One key component is affective sharing, which relies on emotional resonance and perceptual simulation. When this shared emotion is self-oriented, an individual may experience empathic distress, potentially leading to withdrawal from the emotional source. Whereas, knowing that the shared emotion originates from the other (i.e., self-other distinction), one may experience other-oriented empathic concern, motivating individuals to act to alleviate others’ suffering (7, 11). Therefore, self-other distinction and the ability to adopt other people’s perspectives is also crucial for empathy and prosocial behavior (5, 7). Given the positive life outcomes associated with empathy, there has been growing interest in methods to cultivate this capacity (9, 12). This is particularly relevant for individuals who may experience empathy depletion, such as healthcare professionals or social service providers (13–15), as well as for those who may have limited empathy, such as individuals with psychopathic tendencies (16). Various empathy intervention methods have been suggested; however, the effectiveness of these interventions varied, with effect sizes ranging from small to medium, depending on the training methods used and the outcome measures assessed (9, 13, 17, 18). A recent meta-analysis study, in particular, investigated differences in intervention effects across three types of training approaches: focusing on the self, the other, or their social relation (9). Interestingly, the largest effect was found in studies that combined all three approaches. While previous intervention studies have reported some success, these methods also have several limitations. They often require significant effort, pose psychological and emotional challenges, demand high levels of motivation, and involve considerable time commitments (13, 19, 20). In addition, many studies rely on subjective report measures to assess the effectiveness of the interventions as revealed in several meta-analyses (9, 18, 21), which can be subject to biases such as social desirability or inaccuracies in reflecting actual change. These challenges can partly be addressed by employing strategies that engage implicit and automatic processes, as well as by adopting objective measures to assess changes in empathic responses.

One such example is our previous study, where we introduced the implicit compassion promotion task (iCPT) (22). The iCPT was devised based on the principles of cognitive bias modification for interpretation (23), employing experimental contingencies that reinforced empathic responses to distressed others. Specifically, the iCPT requires participants to read textual scenarios featuring distressed individuals, with a fragmented word missing letters in the last sentence. Participants complete the word by filling in missing letters, resulting in words that convey empathic concern toward distressed others. This task is simple and easy to perform, carried out without explicit instructions. However, through repetitive and consistent reinforcement, the iCPT is designed to cultivate a tendency to elicit empathic concern toward those in distress (22). In a series of two experimental examinations, we found that participants who underwent the iCPT intervention exhibited enhanced behavioral and neural responses related to empathy than those who underwent a control version of the iCPT (Kim, Hamann, et al., 2021). While this prior study highlighted the potential of the iCPT, its small sample size limited the generalizability of the results. Furthermore, the original iCPT can be improved by employing gamification elements, which can increase adherence and engagement while reducing boredom during the intervention (24–27).

Following earlier attempts at gamifying implicit interventions (25), we have modified the original iCPT (22) into a typing game format, incorporating game elements such as point systems, time pressure, and adaptiveness. This gamified version of the iCPT, referred to as the gam-iCPT, maintains the original procedures of presenting written scenarios with an incomplete word. Notably, this version introduces two incomplete, fragmented words descending from the top of the screen, with only one target word completing the scenario meaningfully. Users are required to type in a syllable to complete the target word. Points were awarded based on performance, and the descending speed of the words increases with improved performance. The gam-iCPT is expected to show similar effects with the original iCPT while increasing engagement (25).

Empathic concern and caring for others rely on the ability to adopt other-oriented perspective (5, 7, 28, 29). When faced with others’ suffering, individuals may instinctively experience self-oriented feelings of empathic distress (4). However, by taking the perspective of others, the focus shifts from the self to the other, fostering a more other-oriented empathic concern. This shift can potentially motivate individuals to engage in actions to reduce others’ suffering (7, 11). An implicit approach to improving perspective taking has been proposed, targeting visual perspective-taking (VPT; 30). VPT refers to the ability to perceive the external environment from the visual viewpoints of others (31) and is considered to reflect low-level, implicit processes of cognitive perspective-taking (32–34). Recent research has highlighted a close association between VPT and trait empathy (32, 35–37). For instance, individuals with higher trait empathic concern performed better on a VPT task that require resolving visual perspective conflicts between the self and others (36) and showed greater altercentric bias during a similar task (38). Furthermore, individuals with antisocial personality disorder exhibited both decreased empathic concern and greater egocentric bias during a VPT task compared to typical individuals (28). These findings suggest a close link between VPT and empathic concern, implying that interventions designed to enhance other-oriented visual perspective taking may foster empathic concern and facilitate actions to reduce others’ suffering.

Following the framework proposed by Cosmoiu et al. (30), we developed the other-oriented visual perspective-taking task (OVPT) adapting the original VPT assessment task established in a prior study (39). In the original VPT task, participants respond to the number of visual items seen congruently or incongruently between the self-oriented and other-oriented perspectives. Successful responses in incongruent trials requires suppressing the irrelevant perspective (39). The OVPT modifies this original protocol by consistently requiring participants to adopt others’ visual viewpoints rather than their own. This repeated adoption of others’ viewpoints may facilitate other-oriented perspective-taking. As it stands, there is a lack of empirical studies systematically testing the effect of VPT training.

The current study aimed to examine whether the applications of the gam-iCPT and OVPT interventions can alter early attentional processing of socioemotional information and enhance empathic responses toward others. We expected that these two interventions directly and indirectly enhance empathic concern and may alter early cognitive processes of socioemotional information (40, 41). In this study, healthy adult participants were recruited and randomly assigned to one of four intervention groups. Each group received a different combination of the intervention tasks: the effective version of either gam-iCPT, OVPT, both, or their respective control versions. Intervention outcomes were assessed using the dot-probe attention bias task with facial expressions of emotions (42, 43) and the empathy rating task, which assessed empathic concern and helping intentions toward distressed others, developed in our previous research (22). We anticipated that the effective interventions would modulate attentional bias to facial expression of emotions and improve empathic and prosocial responses. We aimed to determine whether the combined intervention would have a greater effect compared to single interventions or a no intervention control.

## 2 Methods

### 2.1 Participants

A total of 128 healthy adults (62 males, mean age = 23.84 ± 3.77) were recruited through an online community for this study. None of the participants had any prior or current neurological or psychiatric illnesses. The sample size was determined based on a standardized medium effect size (44), due to the limited availability of comparable studies. Using Cohen’s *f* of 0.25 along with an alpha level of 0.05 and a power of 0.80, the G*Power software version 3.1.9.4 (45) resulted in a required sample size of 128. An equal number of participants were randomly assigned to each of the four experimental groups. All participants provided written informed consent before participation. This study was approved by the ethics committee of Korea University and conducted following the principles of the Declaration of Helsinki.

### 2.2 Tasks and materials

#### 2.2.1 Intervention task: the gamified implicit compassion promotion task

**Figure 1:**
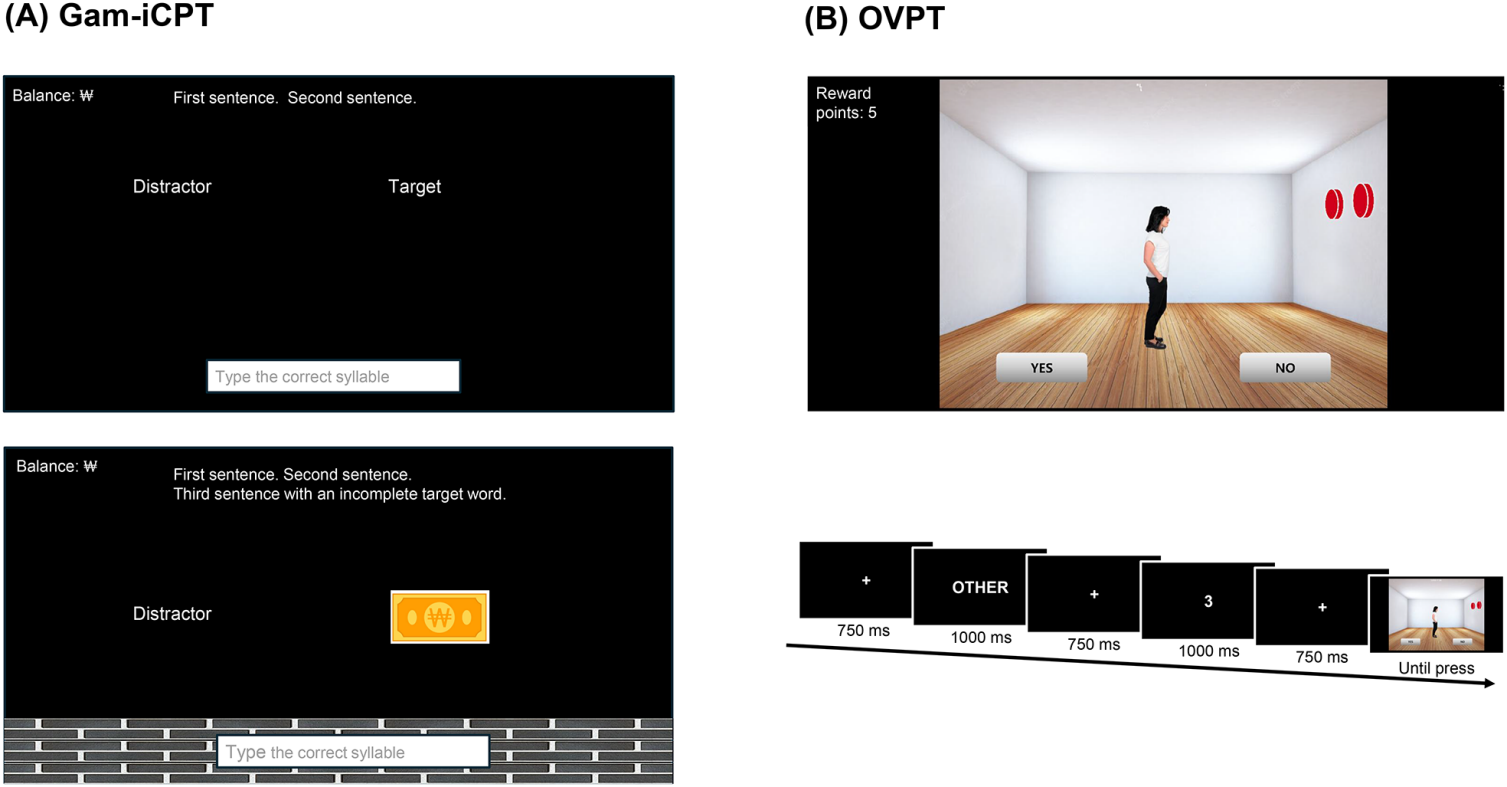
**(A)** Illustration of Example Trials of the gam-iCPT. The lower figure shows the monetary feedback provided for correct responses, along with an elevated floor. **(B)** Illustration of Example Trials of the OVPT.

The gam-iCPT was developed as a web application with a visually engaging background that mimics a typing game (**Figure 1A**). This application was created using the Flutter Software Development Kit (Google, www.flutter.dev) and is hosted on a cloud-based platform via Firebase (Google, firebase.google.com). The gam-iCPT requires participants to complete a fragmented target word by typing the correct letters. The task employed textual scenarios developed in a prior study (22). There were two types of scenarios: empathic and neutral scenarios. Empathic scenarios depicted social interactions featuring a person experiencing a difficult situation, while neutral scenarios depicted ordinary, everyday social interactions. These scenarios were written from a first-person perspective and consisted of three sentences each. In the empathic scenarios, the first sentence introduced a social situation in which the protagonist (“I”) encounters a person facing difficulty. The second sentence described the distress experienced by the target person, and the third sentence depicted the protagonist’s empathic concern for the person. The neutral scenarios followed a similar structure, with the first sentence setting the social context and the second and third sentences detailing the interaction and its resolution.

We prepared two versions of the task: the effective version and control version. These two versions differed in the proportion of scenario types. The effective version included 48 unique empathic scenarios and 13 unique neutral scenarios; while the control version included 13 unique empathic scenarios and 48 unique neutral scenarios. To minimize participants’ explicit awareness of response contingencies, a small number of the contrasting type of scenario (empathic or neutral) was included in each version. In both versions, scenarios were presented in a random order.

During the task, each trial started with the first two sentences sequentially appearing on a computer screen with a duration of 4 seconds each. The last sentence then appeared, containing a missing word. Simultaneously, two fragmented words each missing a phoneme descended from the top of the screen in random zig-zag directions to the bottom of the screen. Only one of these words was the target of the trial and semantically coherent with the scenario. Participants were instructed to type in the missing letters to complete the target word.

The target and distractor words were selected from the standardized word lists, with matching levels of emotional valence and arousal (**Supplementary Table S1**; 46, 47). Target words in the emotional scenarios conveyed empathic concerns (e.g., “concerned,” “worried”), and distractor words connoted general emotional states (“disappointed,” “bored”). In the neutral scenarios, the target and distractor words denoted common objects (e.g., “building,” “table”). Typing correct letters were marked by a monetary symbol appearing on the target word, accompanied by a simple auditory tone. If participants did not complete the word before it reached the floor of the screen or if they provided an incorrect answer, the trial ended and was coded as a miss. After completion of 25 correct trials, the landing point of the words was slightly elevated for the subsequent trials to encourage faster responses (**Figure 1A**).

#### 2.2.2 Intervention task: other-oriented visual perspective-taking task

The OVPT was developed using Flutter and Firebase, employing similar stimuli and procedures as the original VPT assessment task from a prior study (39). The key features of the task included an avatar positioned in the center of a room, facing either the left or right wall. Red discs were displayed on one or both sides of the wall. The number of the discs changed in each trial, ranging from one to three (**Figure 1B**). The task was constructed to introduce inconsistency between the number of discs visible to the participants and those to the avatar.

Two versions of the task were created: the effective version and control version. Each version consisted of two blocks of 52 trials each, totaling 104 trials. The effective version required participants to always take the perspective of the avatar, aiming to induce other-oriented bias; while the control version required participants to take either perspective equally frequently. Each block included four filler trials in which no discs were displayed on either wall, resulting in the disc number 0. The gender of the avatar was matched with that of the participant. Participants performed a practice task of 16 trials before proceeding to the main task.

During the task, each trial began with a fixation cross lasted for 750 ms in the center of the screen. After a blank screen of 1000 ms, the word “YOU” or “OTHER” was displayed for 750 ms to instruct the participant to take their own visual perspective (“YOU”) or that of the avatar (“OTHER”), respectively. After a 1000 ms blank screen, a digit from 0 to 3 was presented for 750 ms to indicate the number of visible discs the participants needed to confirm according to the instructed perspective. Then, the picture of the room, avatar, and discs appeared centrally on screen until a response was made. Participants indicated their responses by pressing the “z” key for a match or the “m” key for a mismatch. The digit was equally frequently matched or mismatched the correct number of discs.

One reward point was accumulated for every correct answer, and the earned points were continually visible in the left top corner of the screen throughout the task. As participants progressed through the task, the response time limit decreased by 1000 ms for every 25 points earned, starting from a default of 8000 ms and reaching a minimum of 4000 ms.

#### 2.2.3 Evaluation task: dot-probe attentional bias task

The materials and procedures for the dot-probe task are detailed in previous studies (22, 48). The task involved 48 pairs of faces: 14 fear-neutral, 14 sad-neutral, 14 happy-neutral, and 6 neutral-neutral pairs. Each pair of faces featured the same face model selected from a standardized database of facial photographs (49).

Each trial began with a fixation point appearing at the center of the screen for 500 ms, followed by the presentation of a pair of faces on the left and right sides of the screen. After 500 ms, the faces disappeared and a small gray dot appeared on either the left or right side of the screen. Participants were instructed to promptly and accurately press either the “z” or “/” key to indicate whether the dot appeared on the left or right side, respectively. The dot remained on the screen until the participant made a response. Participants completed a total of 192 trials, which included four repetitions of each of the 48 unique pairs of faces. A one-minute break was provided halfway through the task.

Two subcomponents of attentional bias were calculated: attentional orienting and disengagement (22, 48). Orienting was determined by calculating the difference in mean reaction times between probes replacing emotional faces in emotional–neutral pairs and probes replacing neutral faces in neutral–neutral pairs. Larger positive orienting scores indicate quicker orienting toward emotional faces. Disengagement was measured by subtracting the mean reaction times to probes replacing neutral faces in neutral–neutral pairs from the mean reaction times to probes presented at the location of the neutral face in emotional–neutral pairs. Larger positive disengagement scores indicate slower disengagement from emotional faces.

#### 2.2.4 Evaluation task: empathy rating task

The empathy rating task was detailed in previous studies (22, 48). This task involved the presentation of short video clips selected from various movies (**Supplementary Table S2**). These clips were categorized into three types: fearful, sad and neutral clips. Fearful clips depicted victims facing panic or immediate threat (e.g., a woman desperately fleeing from danger), while sad clips depicted victims suffering from losses or illness (e.g., a boy experiencing family separation). Neutral clips portrayed individuals engaged in ordinary life events (e.g., a man taking a study break).

A total of 9 video clips (comprising 3 fear, 3 sad, and 3 neutral) were presented in pseudo-random order, so that no clips of the same type were presented consecutively. Each trial began with of a fixation cross for 1 s, followed by a brief written context about the video clip, including information about the target character, relationships between characters, and preceding events, for a duration of 12 s. Subsequently, the video clip was played, and participants were instructed to watch it from the first-person perspective. Following the presentation, participants provided three self-reported ratings sequentially.

Participants rated empathic distress (e.g., how distressed they were about the event involving the victim), empathic concern (e.g., how concerned they were for the victim) and helping intentions (e.g., how much they wanted to help the victim). Participants assigned the ratings on a 7-point Likert scale (1= least distressed/concerned/willingness to help, 7= most distressed/concerned/willingness to help) and pressed the corresponding number on the keyboard. We did not obtain ratings of helping intentions for neutral events as the target character appearing in the neutral events did not apparently need help. After participants provided all ratings in a self-paced manner, there was a 10-sec break before moving to the next video clip.

### 2.3 Procedures

Upon arrival, participants completed written informed consent form and were randomly assigned to one of four groups depending on their assigned intervention tasks. All participants completed self-reported questionnaires including the Positive Affect and Negative Affect Schedule (PANAS; 50), the Spielberger State-Trait Anxiety Inventory (STAI; 51), the Beck Depression Inventory (BDI; 52), and the Interpersonal Reactivity Index (IRI; 4).

Following this, participants were engaged in the intervention tasks. Each group received a different combination of the effective version or the control version of the intervention tasks. The combined group received the effective versions of both the OVPT and gam-iCPT. The OVPT group received the effective version of the OVPT and the control version of the gam-iCPT. The gam-iCPT group received the control version of the OVPT and the effective version of the gam-iCPT. Finally, the control group received the control versions of both the OVPT and gam-iCPT. Overall, the completion of these intervention tasks took approximately less than one hour.

After a short break, participants completed additional self-reported questionnaires, including the Prosocialness Scale for Adults (PSA; 53), the Emotion Regulation Questionnaire (ERQ; 54), and the Adult Emotional Quotient Test (AEQT; 55). Participants then filled out the PANAS once more before proceeding to the outcome measures: the dot-probe task and the empathy rating task. Finally, participants were debriefed and thanked for their participation.

## 3 Result

### 3.1 Demographic and self-reported questionnaires

**Table 1** shows the means and standard deviations of the descriptive variables for each group, along with the results of the group test statistics. One-way ANOVAs for each descriptive variable revealed that groups were similar for age, state and trait anxiety (STAI-S and STAI–T), depression (BDI), trait empathy (IRI), prosocialness (PSA), emotion regulation (ERQ), and emotional intelligence (AEQT) (**Table 1**).

**Table 1:** Descriptive statistics and the results of the group tests.

Measures	Combined (n=32)		gam-iCPT (n=32)		OVPT(n=32)		Control (n=32)		TestStatistics	
	*Mean*	*SD*	*Mean*	*SD*	*Mean*	*SD*	*Mean*	*SD*	*F*	*p*
Age	22.56	2.78	23.63	3.77	24.84	4.52	24.31	3.59	2.24	.09
Gender (males)	16		15		17		14			
STAI - State Anxiety	45.34	1.83	45.16	1.76	45.81	2.68	45.31	2.49	.52	.67
STAI - Trait Anxiety	48.94	2.94	47.88	2.43	48.59	3.02	48.56	3.55	.7	.55
BDI	5.75	4.86	6.53	5.71	6.16	5.30	5.88	6.78	.12	.95
ERQ	32.53	5.41	32.16	4.35	32.09	6.61	33.28	5.66	.31	.82
AEQT	127.97	14.20	124.44	11.49	124.88	14.69	130.75	16.76	1.34	.26
IRI - Fantasy	16.63	4.67	17.34	4.49	16.72	4.45	17.50	4.59	.30	.83
IRI - Perspective Taking	22.19	4.04	21.44	4.21	21.09	3.71	21.53	3.84	.43	.73
IRI - Empathic Concern	24.22	3.57	23.38	4.05	22.69	3.95	23.16	3.28	.94	.42
IRI - Personal Distress	21.38	5.71	21.22	5.10	21.41	5.52	21.44	3.66	.01	1.00
PSA	55.53	9.55	52.38	9.40	51.84	11.78	55.78	10.86	1.25	.30

The combined group received the effective versions of both interventions. The gam-iCPT received the effective gam-iCPT and neutral OVPT. The OVPT received the effective OVPT and neural gam-iCPT. The control group received control versions for both interventions. STAI, Spielberger State-Trait Anxiety Inventory; BDI, Beck Depression Inventory; ERQ, Emotion Regulation Questionnaire; AEQT, Adult Emotional Quotient Test; IRI, Interpersonal Reactivity Index; PSA, Prosocialness Scale for Adults.

Participants completed the intervention tasks with an overall mean accuracy closer to 100% (gam-iCPT, 97.99% ± 2.65; OVPT, 97.96% ± 3.48), which was expected given the ease and simplicity of the tasks.

### 3.2 Outcome measures

**Table 2** presents the means and standard deviations of all outcome measures for each group and condition.

**Table 2:** Means and standard deviations of measures from each outcome test.

Task/Measure		Combined		gam-iCPT		OVPT		Control	
		*Mean*	*SD*	*Mean*	*SD*	*Mean*	*SD*	*Mean*	*SD*
Dot-Probe Attentional Bias Task									
Attentional Orienting	Fearful	5.16	11.77	-0.46	18.38	0.19	23.52	4.58	17.99
	Happy	3.63	14.06	-3.57	15.45	-2.86	24.45	-1.66	17.87
	Sad	4.77	14.28	-2.13	9.32	-6.51	35.55	2.95	24.96
Attentional Disengagement	Fearful	-1.20	17.80	5.17	19.75	5.30	26.19	-1.41	20.95
	Happy	-4.18	17.06	5.55	19.41	-1.06	26.43	-6.49	33.19
	Sad	-5.47	14.89	7.10	21.95	3.31	30.95	-2.28	25.48
Empathy Rating Task									
Empathic Concern	Fearful clip	6.42	0.92	6.31	1.03	6.29	0.65	6.31	0.79
	Sad clip	6.06	0.86	5.60	0.83	5.51	0.88	5.83	1.06
	Neutral clip	3.82	0.94	3.96	0.86	3.86	0.93	2.23	0.87
Empathic Distress	Fearful clip	5.35	1.54	4.96	1.28	5.09	1.39	4.71	1.64
	Sad clip	5.26	1.31	4.61	1.25	4.76	1.46	4.73	1.14
	Neutral clip	2.35	0.68	2.25	0.83	2.25	0.83	2.66	0.44
Helping intentions	Fearful clip	6.52	0.60	6.33	0.92	6.60	0.50	6.39	0.94
	Sad clip	5.63	1.15	4.91	1.02	5.20	0.97	5.29	1.32
Positive and Negative Affect Scale									
Positive Affect	pre	24.50	4.20	21.63	4.46	22.28	4.90	24.38	6.05
	post	24.56	4.49	21.50	4.52	22.81	5.02	24.09	7.00
Negative Affect	pre	20.19	3.84	17.41	3.46	18.69	4.68	19.56	5.01
	post	19.47	4.38	16.81	3.44	18.31	4.04	18.97	5.37

The combined group received the effective versions of both interventions. The gam-iCPT received the effective gam-iCPT and neutral OVPT. The OVPT received the effective OVPT and neural gam-iCPT. The control group received control versions for both interventions.

#### 3.2.1 Dot-probe attention bias task

Overall, a total of 2.87% of the trials in the dot-probe task were excluded due to incorrect (0.56%) and outlier responses (2.31%). Outliers were identified as trials with reaction times either below 200 ms or exceeding 2.5 standard deviations above each participant’s mean reaction time, following previous approach (22, 56).

**Figure 2:**
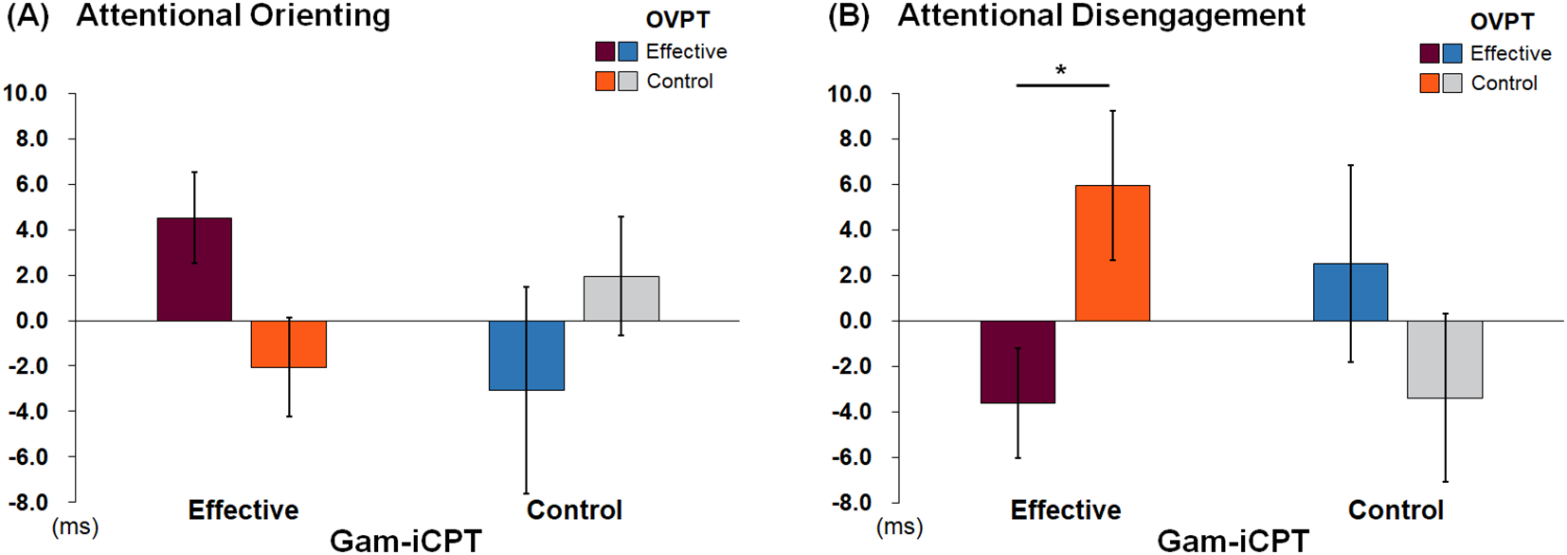
Attentional Orienting **(A)** and Disengagement Scores **(B)** per Group. Error bars represent standard errors. *p = .056.

We performed 3 × 2 × 2 ANOVAs on each measure, with emotion (fearful vs. happy vs. sad) as the within-subjects factor, and gam-iCPT (effective vs. control) and OVPT (effective vs. control) as the between-subjects factors. For orienting, we found no main effects of emotion, gam-iCPT, or OVPT, *F*s < 2.5, *ns*. We also did not observe significant two-way interactions between emotion and gam-iCPT, or emotion and OVPT, *Fs* < 1, nor a significant three-way interaction, *F* < 1. However, a marginal two-way interaction was found between gam-iCPT and OVPT, *F* (1, 124) = 3.73, *p* = .056, *η_p_* ^2^ = .029 (**Figure 2A**). Follow-up simple effects analyses indicated no statistically significant differences between comparisons, *F*s < 3.2, *ns*.

For attentional disengagement, we observed no significant main effects of emotion, gam-iCPT, or OVPT, *F*s < 2. We also did not observe significant two-way interactions between emotion and gam-iCPT, or emotion and OVPT, *F*s < 1, nor a three-way interaction, *F* < 1. However, we found a significant interaction between gam-iCPT and OVPT, *F* (1, 124) = 4.86, *p* = .029, *η_p_* ^2^ = .038 (**Figure 2B**). Follow-up simple effects analyses indicated that, among participants who received the effective gam-iCPT, the effective OVPT (mean = -3.62, sd = 13.69) resulted in faster disengagement compared to the control OVPT (mean = 5.94, sd = 18.579), *F* (1, 124) = 3.71, *p* = .056, *η_p_* ^2^ = .029. In contrast, among participants who received the control gam-iCPT, there was no effect of OVPT, *F* (1, 124) = 1.42, *p* = .24.

#### 3.2.2 Empathy rating task

We conducted 2 × 2 × 2 ANOVAs with emotion (fearful vs. sad) as the within-subjects factor, and gam-iCPT (effective vs. control) and OVPT (effective vs. control) as the between-subjects factors, separately for ratings of empathic concern, empathic distress, and helping intentions.

**Figure 3:**
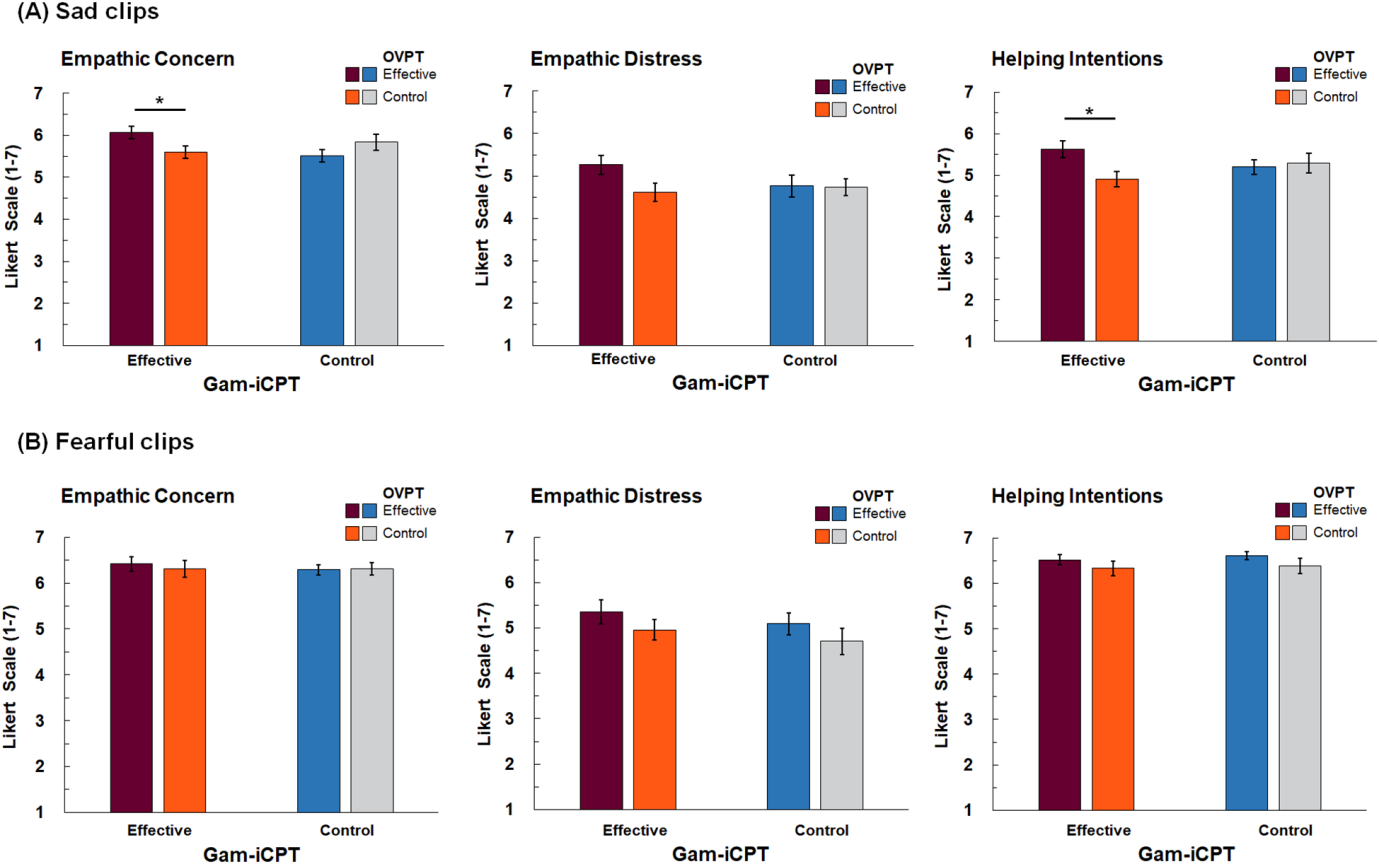
Ratings of Empathic Concern, Empathic Distress, and Helping Intentions for Sad Video Clips **(A)** and Fearful Video Clips **(B)**. Error bars represent standard errors. *p < .05.

For the ratings of empathic concern, there were a significant main effect of emotion, *F* (1, 124) = 60.16, *p* <.001, *η_p_* ^2^ = .33, indicating increased empathic concern for the fearful clips (mean = 6.33, sd = .85) compared to sad clips (mean = 5.75, sd = .93). No other main effects of gam-iCPT, or OVPT were significant, *F* < 1. We also did not observe significant two-way interactions between emotion and gam-iCPT, emotion and OVPT, or OVPT and gam-iCPT, *F*s < 2.75. However, there was a significant three-way interaction, *F* (1, 124) = 4.79, *p* = .031, *η_p_* ^2^ = .037. In order to disentangle the 3-way interaction, separate 2 × 2 ANOVAs were conducted for sad and fearful video clips. For sad video clips, we found a significant interaction between gam-iCPT and OVPT, *F* (1, 124) = 5.87, *p* = .017, *η_p_* ^2^ = .045. Follow-up simple effects analyses indicated that, among participants who received the effective gam-iCPT, those who also received the effective OVPT (mean = 6.06, sd = .86) showed increased empathic concern compared to those who received the control OVPT (mean = 5.60, sd = .83), *F* (1, 124) = 4.04, *p* = .047, *η_p_* ^2^ = .032 (**Figure 3A**). In contrast, among participants who received the control gam-iCPT, there was no significant effect of gam-iCPT, *F* (1, 124) = 2.01, *p* = .16. For fearful video clips, no main effects or interactions were observed, *Fs* < 1.0, *ns*.

For the ratings of empathic distress, there was a marginally significant emotion effect, *F* (1, 124) = 3.47, *p* = .065, *η_p_* ^2^ = .027, indicating a tendency of increased distress for the fearful video clips (mean = 5.03, sd = 1.47) compared to sad clips (mean = 4.84, sd = 1.30). No other statistically significant main effects of gam-iCPT or OVPT were significant, *Fs* < 2.7, *ps* >.1. We also did not observe significant two-way interactions between emotion and gam-iCPT, emotion and OVPT, or OVPT and gam-iCPT, *Fs* < 1, nor a significant three-way interaction, *F* < 2.25.

For helping intentions ratings, there was a main effect of emotion, *F* (1, 124) = 181.96, *p* <.001, *η_p_* ^2^ = .595, indicating increased helping intentions for the fearful clips (mean = 6.46, sd = .76) compared to sad clips (mean = 5.26, sd = 1.14). No other main effects of gam-iCPT, or OVPT were significant, *F*s < 1. We also did not observe significant two-way interactions between emotion and gam-iCPT, emotion and OVPT, or OVPT and gam-iCPT, *F*s < 2, *ps* >.15. However, we observed a significant three-way interaction, *F* (1, 124) = 5.57, *p* = .020, *η_p_* ^2^ = .043. To further examine the interaction, 2 × 2 ANOVAs were conducted separately for fearful and sad video clips. For sad clips, there was significant interaction between gam-iCPT and OVPT, *F* (1, 124) = 4.19, *p* = .043, *η_p_* ^2^ = .033. Follow-up simple effects analyses indicated that, among participants who received the effective gam-iCPT, those who also received the effective OVPT (mean = 5.63, sd = 1.15) reported increased helping intentions compared to those who received the control OVPT (mean = 4.91 ms, sd = 1.02), *F* (1, 124) = 6.55, *p* = .012, *η_p_* ^2^ = .050 (**Figure 3C**). In contrast, among participants who received the control gam-iCPT, there was no effect of the OVPT, *F* (1, 124) = 0.11, *ns*. For fearful clips, no significant main or interaction effects were observed, *Fs* < 2.5, *ps* >.1.

#### 3.2.3 Mood changes

In order to examine whether there were significant positive or negative mood changes following the intervention treatment, we conducted 2 × 2 ANOVAs with gam-iCPT (effective vs. control) and OVPT (effective vs. control) as the between-subjects factors separately for positive and negative mood change scores. Mood change scores were calculated by subtracting pre-intervention PANAS positive (or negative) affect scores from those of post-intervention scores. There were no significant main effects or interaction effects for positive mood change scores, *F*s<1, *ns*, and negative mood change scores, *F*s<.2, *ns*, indicating that interventions did not alter mood.

## 4 Discussion

In the current study we examined whether interventions designed to promote visual perspective-taking and empathic concern influence early attentional processes of socioemotional information, as well as empathic responses. Differences in outcome measures were consistently observed between the combined intervention and the single intervention of gam-iCPT. Specifically, participants treated with both the effective gam-iCPT and effective OVPT showed faster disengagement from emotional faces, as well as greater empathic concern and increased helping intentions toward sad victims, compared to those treated with the effective gam-iCPT and control OVPT. These results suggest the promising potential of the combined interventions. However, it is also notable that neither intervention individually produced a significant effect in the study. This lack of main effects for each intervention strategy highlight the need for caution in interpreting the findings of the current study.

The combined intervention effect, particularly when compared to the effective gam-iCPT intervention, on attentional disengagement and helping intentions may be associated with enhanced top-down control over emotional disturbance. That is, while early attentional orienting is driven by bottom-up factors such as the salience of emotional distractors, disengaging attention from these emotional distractors often requires top-down inhibition and goal-driven selection (57–59). In the dot-probe task, emotional faces may initially attract attentional resources against neutral faces; thus, a prompt response to the target requires top-down attentional control over the distracting emotional information. Similarly, engaging in helping behavior to alleviate others’ suffering requires cognitive control to regulate empathic overarousal and personal distress in response to others’ suffering (60–62). This cognitive control could have been strengthened through the OVPT, which required participants to consistently engage in other-oriented visual perspective taking while suppressing interference from their own perspective. Previous evidence from cognitive neuroscience research suggests that perspective taking relies on prefrontal resources involved in executive control. That is, adopting the perspective of others increased activation in the prefrontal cortex (63, 64) and damage to the frontal cortex impaired performance on tasks requiring perspective-taking (65). Furthermore, noninvasive brain stimulation over the dorsolateral prefrontal cortex improved performance on these tasks (66). When the OVPT was combined with the gam-iCPT, which continuously exposed participants to others’ emotional pain, it may have led to a more effective executive control for subsequently experienced empathic distress, which, in turn, resulted in increased empathic and prosocial responses (60). The importance of goal-directed attention and executive control is further supported by recent findings, showing that violent offenders, who are often characterized by a lack of empathy, made more errors during a visual search task when distractors were present (67).

We did not observe the overall main effects of the gam-iCPT, which was unexpected considering that the original version (iCPT) yielded significant results in a previous study (22). Specifically, healthy participants who underwent the original iCPT exhibited a decrease in empathic distress and reduced attentional orienting to fearful faces compared to the control group (22). In contrast, the current study found that the gam-iCPT intervention reveal comparable results to the control intervention. Given that the current study applied two separate interventions, either the effective or the control version, the null effect may be the due to a complex interaction between these two types, which could not be addressed within the scope of this study. Alternatively, we speculate the possibility that the previous findings of decreases in empathic distress and reduced attentional orienting could be related to empathy fatigue resulting from the repeated empathic engagement during iCPT training (68). That is, participants receiving the iCPT may have developed a defensive tendency to avoid emotionally charged stimuli to protect themselves from psychological pain. This is somewhat similar to the way meditation based mindfulness or compassion training can initially diminish positive affect (69), possibly because early experiences of such training may be emotionally challenging (19, 70, 71).

In light of this view, the provision of the OVPT in addition to the gam-iCPT in the current combined approach may have facilitated participants’ shifts in perspective, helping them recognize that the source of distress lies in others, thereby reducing empathy fatigue. This, in turn, could potentially have led to an increase in empathic concern and helping intentions for the sad victims in the combined intervention group compared to the gam-iCPT intervention group. This view aligns with previous findings that medical students exhibited greater empathic concern for patients when actively adopting patients’ perspectives rather than medical professionals’ perspectives (72), and that their self-reported perspective-taking predicted higher levels of empathy as physicians (73).It is interesting to note that our findings of combined intervention effects on empathic response depended on the type of distressful situations. Specifically, increased empathic concern and helping intentions were prominent for victims depicted in the sad video clips, but not for victims depicted in fearful video clips. Although both fear and sadness are withdrawal-related aversive emotions, and insensitivity to them has been implicated in empathy deficits (74–76), they differ in several psychological and neurobiological bases (77, 78). Fear is reaction to imminent threat and characterized by heightened physiological arousal that facilitates urgent avoidance. In contrast, sadness is reaction to losses and characterized by lower physiological arousal that helps conserve resources (78). Thus, different approaches and resources may be required for coping with these two emotions. While fear often demands immediate, active bodily reactions, sadness typically require more psychological and emotional support (79). Therefore, our results of intervention effects limited to victims experiencing sad events might be related to the nature of intervention training, which focused on the psychological capacities rather than physical ones. Alternatively, the severity of the emotional distress depicted in the video clips may have influenced empathic and prosocial reactions to the victims. That is, fearful information is typically experienced psychologically and physiologically more intense than sad information (78, 80). Therefore, observing others in fearful events may trigger motivational withdrawal due to emphatic distress, resulting in a dissociation of intervention effects across sad and fearful video clips. Further research is warranted to address effective empathy interventions tailored to different types of emotional distress.

Although this study reports intervention effects on attention processes and empathic responses, these effects were limited to the combined intervention group in comparison to the single gam-iCPT intervention group. No significant main effects were observed for either the gam-iCPT or OVPT. This suggests that, in their current form, the interventions may not be sufficiently robust or may require further refinement to produce measurable impacts on the targeted socioemotional outcomes. These findings highlight the complexity of psychological mechanisms underlying empathy intervention. Given these findings, future studies could benefit from employing multimodal approaches that integrates neural, cognitive, and experiential measures. Such an approach would provide a more comprehensive understanding of the underlying psychological mechanisms and could inform the development of more effective interventions.

Another limitation of the current study, which partly related to the above issue, regards the duration of the intervention. Participants underwent only a single training session which lasted less than an hour. This single engagement with intervention tasks may not have been sufficient for the effects to fully manifest, especially considering the initial psychological and emotional load that participants may experience as they begin the intervention tasks (19, 69–71). To explain, relative to the control versions, the effective versions of the gam-iCPT and OVPT involve more frequent exposure to emotionally distressing materials and greater demands for top-down control, which may temporarily deplete cognitive resources. These initial adaptation demands could have challenged participants’ test performance. Extending the intervention to multiple sessions may provide participants with sufficient time to gradually adapt to the demands, potentially leading to more favorable effects. Indeed, a recent meta-analysis of empathy training studies revealed that longer training time was positively associated with the intervention effects, as measured by self-reported empathy scales (9). However, it is also worth noting that meta-analyses on the impact of the number of training sessions on the effectiveness of implicit task-based interventions, such as those used in the current study, have shown mixed results, with some reporting positive effects (81) and others finding no effects (82).

Since we adopted a one-day, single training session, the outcome measures were assessed once after the interventions, which we consider another limitation of this study. The rationale for this approach was to prevent any confounding effects from task repetition within a short time frame. Provided that we observed no group differences in trait levels of empathy, prosociality, emotional intelligence, or emotion regulation styles, which could be associated with individual differences in performance on the outcome tests, we assumed similar socioemotional characteristics and baseline abilities across all groups. Therefore, any group differences were attributed to intervention treatment. However, without a pretest-posttest design, the precise effects of the interventions, as well as individual differences in sensitivity to them, may not be fully assessed. Additionally, the study lacks follow-up assessments, limiting our ability to evaluate the long-term effects of the interventions. Therefore, future research could consider incorporating baseline assessments before the interventions and utilizing larger sample sizes to better explore how individual differences contribute to and interact with intervention outcomes, as well as assess the sustainability of the intervention effects over time.

Despite these limitations, the findings of the current study point to the possibility of the combined intervention. Building upon our previous work on implicit intervention (22), we introduced two implicit intervention strategies to cultivate empathic concern and prosocial responses. Although findings of the current study are limited, they highlight the potential of the combined approach. Future studies may consider employing multisession training, pre- and post-intervention assessments, and multimodal approaches to improve efficacy and better understand how these interventions work.

## Data Availability

The raw data supporting the conclusions of this article will be made available by the authors, without undue reservation.

## References

[1] Decety J Bartal IBA Uzefovsky F Knafo-Noam A. Empathy as a driver of prosocial behaviour: highly conserved neurobehavioural mechanisms across species. Philos Trans R Soc B Biol Sci. (2016) 371:20150077. doi: 10.1098/rstb.2015.0077 PMC4685523 26644596

[2] Eisenberg N Fabes RA Spinrad TL. Prosocial development. In: Eisenberg N, editor. Handbook of child psychology: social, emotional, and personality development, vol. 3 . Willey (2006). p. 646–718. doi: 10.1002/9780470147658.chpsy0311

[3] Grühn D Rebucal K Diehl M Lumley M Labouvie-Vief G. Empathy across the adult lifespan: Longitudinal and experience-sampling findings. Emotion. (2008) 8:753. doi: 10.1037/a0014123 19102586 PMC2669929

[4] Davis MH. A multidimensional approach to individual differences in empathy. JSAS Catal. Select. Doc Psychol. (1980) 10:85.

[5] Shamay-Tsoory SG. The neural bases for empathy. Neuroscientist. (2011) 17:18–24. doi: 10.1177/1073858410379268 21071616

[6] Decety J Yoder KJ. Empathy and motivation for justice: Cognitive empathy and concern, but not emotional empathy, predict sensitivity to injustice for others. Soc Neurosci. (2016) 11:1–14. doi: 10.1080/17470919.2015.1029593 25768232 PMC4592359

[7] Lamm C Rütgen M Wagner IC. Imaging empathy and prosocial emotions. Neurosci Lett. (2019) 693:49–53. doi: 10.1016/j.neulet.2017.06.054 28668381

[8] Singer T Klimecki OM. Empathy and compassion. Curr Biol. (2014) 24:R875–8. doi: 10.1016/j.cub.2014.06.054 25247366

[9] Wu X Yao SC Lu XJ Zhou YQ Kong YZ Hu L. Categories of training to improve empathy: A systematic review and meta-analysis. Psychol Bull. (2024) 150:1237. doi: 10.1037/bul0000453 39418441

[10] Zaki J Ochsner KN. The neuroscience of empathy: progress, pitfalls and promise. Nat Neurosci. (2012) 15:675–80. doi: 10.1038/nn.3085 22504346

[11] Batson C. These things called empathy: eight related but distinct phenomena. In: Decety J Ickes W, editors. The social neuroscience of empathy. MIT Press (2009). doi: 10.7551/mitpress/9780262012973.003.0002

[12] Davis MH Begovic E. Empathy-related interventions. In: Parks AC Schueller SM, editors. The wiley blackwell handbook of positive psychological interventions. Wiley-Blackwell (2014). p. 111–34. doi: 10.1002/9781118315927.ch6

[13] Chen X Chen M Zheng H Wang C Chen H Wu Q. Effects of psychological intervention on empathy fatigue in nurses: A meta-analysis. Front Public Health. (2022) 10:952932. doi: 10.3389/fpubh.2022.952932 36311568 PMC9614432

[14] Levett-Jones T Cant R Lapkin S. A systematic review of the effectiveness of empathy education for undergraduate nursing students. Nurse Educ Today. (2019) 75:80–94. doi: 10.1016/j.nedt.2019.01.006 30739841

[15] Paulus CM Meinken S. The effectiveness of empathy training in health care: a meta-analysis of training content and methods. Int J Med Educ. (2022) 13:1. doi: 10.5116/ijme.61d4.4216 35092671 PMC8995011

[16] Blair RJR. Traits of empathy and anger: implications for psychopathy and other disorders associated with aggression. Philos Trans R Soc B Biol Sci. (2018) 373:20170155. doi: 10.1098/rstb.2017.0155 PMC5832681 29483341

[17] Luberto CM Shinday N Song R Philpotts LL Park ER Fricchione GL. A systematic review and meta-analysis of the effects of meditation on empathy, compassion, and prosocial behaviors. Mindfulness. (2018) 9:708–24. doi: 10.1007/s12671-017-0841-8 PMC6081743 30100929

[18] van Berkhout ET Malouff J. The efficacy of empathy training: a meta-analysis of randomized controlled trials. J Couns. Psychol. (2016) 63:32–41. doi: 10.1037/cou0000093 26191979

[19] Crane C Williams JMG. Factors associated with attrition from mindfulness-based cognitive therapy in patients with a history of suicidal depression. Mindfulness. (2010) 1:10–20. doi: 10.1007/s12671-010-0003-8 21125023 PMC2987524

[20] Lumma A-L Kok BE Singer T. Is meditation always relaxing? Investigating heart rate, heart rate variability, experienced effort and likeability during training of three types of meditation. Int J Psychophysiol. (2015) 97:38–45. doi: 10.1016/j.ijpsycho.2015.04.017 25937346

[21] Winter R Issa E Roberts N Norman RI Howick J. Assessing the effect of empathy-enhancing interventions in health education and training: a systematic review of randomised controlled trials. BMJ Open. (2020) 10:e036471. doi: 10.1136/bmjopen-2019-036471 PMC7520826 32978187

[22] Kim SA Hamann S Kim SH. Neurocognitive mechanisms underlying improvement of prosocial responses by a novel implicit compassion promotion task. NeuroImage. (2021) 240:118333. doi: 10.1016/j.neuroimage.2021.118333 34229063

[23] MacLeod C Mathews A. Cognitive bias modification approaches to anxiety. Annu Rev Clin Psychol. (2012) 8:189–217. doi: 10.1146/annurev-clinpsy-032511-143052 22035241

[24] Hamari J Koivisto J Sarsa H. Does gamification work?- a literature review of empirical studies on gamification, in: 2014 47th Hawaii international conference on system sciences. IEEE. (2014), 3025–34. doi: 10.1109/HICSS.2014.377

[25] Salemink E de Jong SR Notebaert L MacLeod C Van Bockstaele B. Gamification of cognitive bias modification for interpretations in anxiety increases training engagement and enjoyment. J Behav Ther Exp Psychiatry. (2022) 76:101727. doi: 10.1016/j.jbtep.2022.101727 35217211

[26] Vermeir JF White MJ Johnson D Crombez G Van Ryckeghem DM. The effects of gamification on computerized cognitive training: systematic review and meta-analysis. JMIR Serious Games. (2020) 8:e18644. doi: 10.2196/18644 32773374 PMC7445616

[27] Vermeir JF White MJ Johnson D Crombez G Van Ryckeghem DM. Gamified web-delivered attentional bias modification training for adults with chronic pain: protocol for a randomized, double-blind, placebo-controlled trial. JMIR Res Protoc. (2022) 11:e32359. doi: 10.2196/32359 35084344 PMC8943713

[28] Bigot A Tiberi LA Saloppé X Nandrino JL Pham T Bukowski H. Confusing my viewpoint with his: Altered self–other distinction performance in antisocial personality disorder. Pers. Disord.: Theory Res Treat. (2025) 16:110–21. doi: 10.1037/per0000660 39760725

[29] Santiesteban I White S Cook J Gilbert SJ Heyes C Bird G. Training social cognition: from imitation to theory of mind. Cognition. (2012) 122:228–35. doi: 10.1016/j.cognition.2011.11.004 22133627

[30] Cosmoiu AM Nedelcea C Podina IR. A computerized, gamified intervention training visual perspective-taking. Theoretical rationale and proposal of a randomized controlled trial. eLearning Softw Educ Conf. (2019) 1:91–7. doi: 10.12753/2066-026X-19-011

[31] Flavell JH Everett BA Croft K Flavell ER. Young children’s knowledge about visual perception: Further evidence for the Level 1–Level 2 distinction. Dev Psychol. (1981) 17:99. doi: 10.1037/0012-1649.17.1.99

[32] Doi H Kanai C Tsumura N Shinohara K Kato N. Lack of implicit visual perspective taking in adult males with autism spectrum disorders. Res Dev Disabil. (2020) 99:103593. doi: 10.1016/j.ridd.2020.103593 32035319

[33] Furlanetto T Becchio C Samson D Apperly I. Altercentric interference in level 1 visual perspective taking reflects the ascription of mental states, not submentalizing. J Exp Psychol Hum Percept. Perform. (2016) 42:158. doi: 10.1037/xhp0000138 26389611

[34] Schurz M Aichhorn M Martin A Perner J. Common brain areas engaged in false belief reasoning and visual perspective taking: a meta-analysis of functional brain imaging studies. Front Hum Neurosci. (2013) 7:712. doi: 10.3389/fnhum.2013.00712 24198773 PMC3814428

[35] Bukowski H Kamal NFA Bennett D Rizzo G O’Tuathaigh C. Association between dispositional empathy and self-other distinction in Irish and Belgian medical students: a cross-sectional analysis. BMJ Open. (2021) 11:e048597. doi: 10.1136/bmjopen-2020-048597 PMC8442071 34521665

[36] Mattan BD Rotshtein P Quinn KA. Empathy and visual perspective-taking performance. Cogn. Neurosci. (2016) 7:170–81. doi: 10.1080/17588928.2015.1085372 26745546

[37] Tomei A Besson J Grivel J. Linking empathy to visuospatial perspective-taking in gambling addiction. Psychiatry Res. (2017) 250:177–84. doi: 10.1016/j.psychres.2016.12.061 28161613

[38] Nielsen MK Slade L Levy JP Holmes A. Inclined to see it your way: Do altercentric intrusion effects in visual perspective taking reflect an intrinsically social process? Q J Exp Psychol. (2015) 68:1931–51. doi: 10.1080/17470218.2015.102320 25849956

[39] Samson D Apperly IA Braithwaite JJ Andrews BJ Bodley Scott SE. Seeing it their way: evidence for rapid and involuntary computation of what other people see. J Exp Psychol Hum Percept. Perform. (2010) 36:1255. doi: 10.1037/a0018729 20731512

[40] Preckel K Kanske P Singer T. On the interaction of social affect and cognition: empathy, compassion and theory of mind. Curr Opin Behav Sci. (2018) 19:1–6. doi: 10.1016/j.cobeha.2017.07.010

[41] Van Overwalle F Baetens K. Understanding others’ actions and goals by mirror and mentalizing systems: a meta-analysis. Neuroimage. (2009) 48:564–84. doi: 10.1016/j.neuroimage.2009.06.009 19524046

[42] Koster EH Crombez G Verschuere B De Houwer J. Selective attention to threat in the dot probe paradigm: Differentiating vigilance and difficulty to disengage. Behav Res Ther. (2004) 42:1183–92. doi: 10.1016/j.brat.2003.08.001 15350857

[43] Posner MI. Orienting of attention. Q J Exp Psychol. (1980) 32:3–25. doi: 10.1080/00335558008248231 7367577

[44] Beck TW. The importance of a *priori* sample size estimation in strength and conditioning research. J Strength Cond. Res. (2013) 27:2323–37. doi: 10.1519/jsc.0b013e318278eea0 23880657

[45] Faul F Erdfelder E Lang A-G Buchner A. G* Power 3: A flexible statistical power analysis program for the social, behavioral, and biomedical sciences. Behav Res Methods. (2007) 39:175–91. doi: 10.3758/BF03193146 17695343

[46] Hong Y Nam Y-e Lee Y. Developing Korean affect word list and it’s application. Korean J Cogn. Sci. (2016) 27:377–406. doi: 10.19066/cogsci.2016.27.3.002

[47] Park I-J Min K-H. Making a list of korean emotion terms and exploring dimensions underlying them. Korean J Soc Pers. Psychol. (2005) 19:109–29.

[48] Lee S Kim SH. Inducing sad recognition bias: A novel emotional probabilistic reward task and its affective consequences. PloS One. (2024) 19:e0291979. doi: 10.1371/journal.pone.0291979 39509427 PMC11542835

[49] Lee T Lee K Lee K Choi J Kim H. The korea university facial expression collection: KUEFC. Korea University, Seoul Korea: Lab. of Behavioral Neuroscience, Dept. of Psychology (2006).

[50] Watson D Clark LA Tellegen A. Development and validation of brief measures of positive and negative affect: the PANAS scales. J Pers. Psychol. (1988) 54:1063–70. doi: 10.1037/0022-3514.54.6.1063 3397865

[51] Spielberger C. Professional manual for the state-trait anger expression inventory. Tampa, FL: University of South Florida (1988). 12.

[52] Beck AT Steer RA Brown G. Beck depression inventory manual. 2nd ed. San Antonio, TX: Psychological Corporation (1996).

[53] Caprara GV Steca P Zelli A Capanna C. A new scale for measuring adults’ prosocialness. Eur J Psychol Assess. (2005) 21:77–89. doi: 10.1027/1015-5759.21.2.77

[54] Gross JJ John OP. Individual differences in two emotion regulation processes: Implications for affect, relationships, and well-being. J Pers. Soc Psychol. (2003) 85:348–62. doi: 10.1037/0022-3514.85.2.348 12916575

[55] Salovey P Mayer JD. Emotional intelligence. Imagin. Cogn. Pers. (1990) 9:185–211. doi: 10.2190/DUGG-P24E-52WK-6CD

[56] Price RB Kuckertz JM Siegle GJ Ladouceur CD Silk JS Ryan ND. Empirical recommendations for improving the stability of the dot-probe task in clinical research. Psychol Assess. (2015) 27:365. doi: 10.1037/pas0000036 25419646 PMC4442069

[57] Gaspelin N Luck SJ. The role of inhibition in avoiding distraction by salient stimuli. Trends Cogn. Sci. (2018) 22:79–92. doi: 10.1016/j.tics.2017.11.001 29191511 PMC5742040

[58] Sanchez A Vanderhasselt MA Baeken C De Raedt R. Effects of tDCS over the right DLPFC on attentional disengagement from positive and negative faces: an eye-tracking study. Cogn. Affect. Behav Neurosci. (2016) 16:1027–38. doi: 10.3758/s13415-016-0450-3 27495805

[59] Theeuwes J. Top–down and bottom–up control of visual selection. Acta Psychol. (2010) 135:77–99. doi: 10.1016/j.actpsy.2010.02.006 20507828

[60] Decety J Lamm C. Human empathy through the lens of social neuroscience. Sci World J. (2006) 6:1146–63. doi: 10.1100/tsw.2006.221 PMC5917291 16998603

[61] Li J Chen Y Lu J Li W Zhen S Zhang D. Does self-control promote prosocial behavior? Evidence from a longitudinal tracking study. Children. (2022) 9:854. doi: 10.3390/children9060854 35740790 PMC9221881

[62] Osgood JM Muraven M. Self-control depletion does not diminish attitudes about being prosocial but does diminish prosocial behaviors. Basic Appl Soc Psychol. (2015) 37:68–80. doi: 10.1080/01973533.2014.996225

[63] Decety J Jackson PL. The functional architecture of human empathy. Behav Cogn. Neurosci Rev. (2004) 3:71–100. doi: 10.1177/1534582304267187 15537986

[64] Healey ML Grossman M. Cognitive and affective perspective-taking: evidence for shared and dissociable anatomical substrates. Front Neurol. (2018) 9:491. doi: 10.3389/fneur.2018.00491 29988515 PMC6026651

[65] Stuss DT Gallup GG Jr. Alexander MP. The frontal lobes are necessary for theory of mind. Brain. (2001) 124:279–86. doi: 10.1093/brain/124.2.279 11157555

[66] Qureshi AW Bretherton L Marsh B Monk RL. Stimulation of the dorsolateral prefrontal cortex impacts conflict resolution in Level-1 visual perspective taking. Cogn. Affect. Behav Neurosci. (2020) 20:565–74. doi: 10.3758/s13415-020-00786-5 PMC7266805 32378060

[67] Slotboom J Hoppenbrouwers SS Bouman YH In’t Hout W Sergiou C van der Stigchel S. Visual attention in violent offenders: susceptibility to distraction. Psychiatry Res. (2017) 251:281–6. doi: 10.1016/j.psychres.2017.02.031 28222312

[68] Figley CR. Compassion fatigue: Psychotherapists’ chronic lack of self care. J Clin Psychol. (2002) 58:1433–41. doi: 10.1002/jclp.10090 12412153

[69] Fredrickson BL Cohn MA Coffey KA Pek J Finkel SM. Open hearts build lives: positive emotions, induced through loving-kindness meditation, build consequential personal resources. J Pers. Soc Psychol. (2008) 95:1045. doi: 10.1037/a0013262 18954193 PMC3156028

[70] Barnhofer T Chittka T Nightingale H Visser C Crane C. State effects of two forms of meditation on prefrontal EEG asymmetry in previously depressed individuals. Mindfulness. (2010) 1:21–7. doi: 10.1007/s12671-010-0004-7 PMC2987525 21125024

[71] Creswell JD. Mindfulness interventions. Annu Rev Psychol. (2017) 68:491–516. doi: 10.1146/annurev-psych-042716-051139 27687118

[72] Kim SA Lee Y-M Hamann S Kim SH. Differences in empathy toward patients between medical and nonmedical students: an fMRI study. Adv Health Sci Educ. (2021) 26:1207–27. doi: 10.1007/s10459-021-10045-y PMC8056797 33877486

[73] Yu J Lee S Kim M Lim K Chang K Lee M. Relationships between perspective-taking, empathic concern, and self-rating of empathy as a physician among medical students. Acad Psychiatry. (2020) 44:316–9. doi: 10.1007/s40596-019-01114-x PMC7237518 31873921

[74] Blair RJR Colledge E Murray L Mitchell D. A selective impairment in the processing of sad and fearful expressions in children with psychopathic tendencies. J Abnorm. Child Psychol. (2001) 29:491–8. doi: 10.1023/A:1012225108281 11761283

[75] Marsh AA Blair RJR. Deficits in facial affect recognition among antisocial populations: A meta-analysis. Neurosci Biobehav. Rev. (2008) 32:454–65. doi: 10.1016/j.neubiorev.2007.08.003 PMC2255599 17915324

[76] Sommer M Hajak G Döhnel K Schwerdtner J Meinhardt J Müller JL. Integration of emotion and cognition in patients with psychopathy. Prog Brain Res. (2006) 156:457–66. doi: 10.1016/S0079-6123(06)56025-X 17015096

[77] Ekman P Levenson RW Friesen WV. Autonomic nervous system activity distinguishes among emotions. Science. (1983) 221:1208–10. doi: 10.1126/science.6612338 6612338

[78] Kreibig SD Wilhelm FH Roth WT Gross JJ. Cardiovascular, electrodermal, and respiratory response patterns to fear-and sadness-inducing films. Psychophysiol. (2007) 44:787–806. doi: 10.1111/j.1469-8986.2007.00550.x 17598878

[79] Charles ST Carstensen LL McFall RM. Problem-solving in the nursing home environment: Age and experience differences in emotional reactions and responses. J Clin Geropsychol. (2001) 7:319–30. doi: 10.1023/A:1011352326374

[80] Russell JA. A circumplex model of affect. J Pers. Soc Psychol. (1980) 39:1161–78. doi: 10.1037/h0077714

[81] Menne-Lothmann C Viechtbauer W Höhn P Kasanova Z Haller SP Drukker M. How to boost positive interpretations? A meta-analysis of the effectiveness of cognitive bias modification for interpretation. PloS One. (2014) 9:e100925. doi: 10.1371/journal.pone.0100925 24968234 PMC4072710

[82] Martinelli A Grüll J Baum C. Attention and interpretation cognitive bias change: A systematic review and meta-analysis of bias modification paradigms. Behav Res Ther. (2022) 157:104180. doi: 10.1016/j.brat.2022.104180 36037642

