# Supplementary material for: Implicit intervention approach for empathy: exploring the combined effects of empathic concern and visual perspective-taking
Source: Front Psychiatry. 2025 May 30;16:1530532. doi: 10.3389/fpsyt.2025.1530532 (PMC12163042; doi:10.3389/fpsyt.2025.1530532)
Supplement: Supplementary file 1 [file Table1.docx]

Table S1

Mean Ratings and Standard Deviations for Target and Distractor Words in Empathy and Neutral Scenarios

|  | Empathy Scenarios  (Park & Min, 2005) | | |  | Neutral Scenarios  (Hong et al., 2016) | | |
| --- | --- | --- | --- | --- | --- | --- | --- |
|  | Target | Distractor | *Comparison test results* |  | Target | Distractor | *Comparison test results* |
| Emotional Valence^*^ | 3.09 (±0.58) | 3.28 (±0.78) | *p= .395* | Emotional Valence^†^ | 5.71(±0.85) | 5.79(±0.94) | *p= .748* |
| Emotional Arousal^*^ | 3.61 (±0.39) | 3.93 (±0.88) | *p= .154* | Emotional Arousal^†^ | 4.02(±0.67) | 4.18(±0.79) | *p= .406* |
| Note. ^*^ 7-point Likert scale (1: negative, low – 7: positive, high), ^†^ 9-point Likert scale (1: negative, low – 9: positive, high) | | | | | | | |

References

Hong, Y., Nam, Y.-e., & Lee, Y. (2016). Developing Korean affect word list and it's application. *Korean Journal of Cognitive Science, 27*(3), 377-406.

Park, I.-J., & Min, K.-H. (2005). Making a List of Korean Emotion Terms and Exploring Dimensions Underlying Them. *Korean Journal of Social and Personality Psychology, 19*(1), 109-129.
