# Supplementary material for: Implicit intervention approach for empathy: exploring the combined effects of empathic concern and visual perspective-taking
Source: Front Psychiatry. 2025 May 30;16:1530532. doi: 10.3389/fpsyt.2025.1530532 (PMC12163042; doi:10.3389/fpsyt.2025.1530532)
Supplement: Supplementary file 2 [file Table2.docx]

Table S2

Background Information and Content of Video Clips Used in the Empathy Rating Task

**Fear Condition**

|  | Title | Year | Director | Background information | Content of video clip |
| --- | --- | --- | --- | --- | --- |
| 1 | Blind | 2011 | Ahn, Sang-hoon | To force her troubled younger brother to go home, a policewoman handcuffed him and tied him to the handle of the passenger seat of the car. However, after a car accident on the bridge, the woman was thrown out of the car, and the car was about to fall into the river. | Lying in the road, the woman struggles to wake up, as her younger brother is tied to the car that is on the verge of falling under a bridge. |
| 2 | Rainbow Eyes | 2007 | Yang, Yun-ho | On a dark night, a woman ran away from someone, driving hurriedly down on a deserted mountain road. While driving down a narrow stretch, she saw a deer in the road and turned the steering wheel so quickly that her car flew off the road. | The woman’s out-of-control car plows through a forest and nearly falls off a cliff. |
| 3 | Voice | 2017 | Lee, Chan-ho et al. | A female social worker was personally investigating the disappearance of her coworker. One day on her way to her husband’s workplace, the woman was chased by a man believed to be related to the case under investigation. | The woman is eventually caught by the man and is on the verge of being hit in the head with a blunt weapon. |

**Sad Condition**

|  | Title | Year | Director | Background information | Content of video clip |
| --- | --- | --- | --- | --- | --- |
| 1 | Set Me Free | 2013 | Kim, Tae-yong | A high school boy had grown up in a youth shelter due to his family’s financial situation. However, because his brother needed to enter the shelter, someone else had to leave. When the head of the shelter tried to force the grown boy out, he knelt down to beg. | The boy cries for mercy, grabbing the shelter director’s leg. |
| 2 | The Preparation | 2017 | Cho, Young-jun | Despite being poor, a woman lived happily with her 30-year-old son with a development disability. However, recently she was diagnosed with a stage 3 brain tumor. She was extremely worried about her son who would be left behind. Therefore, she prayed in a church that she usually did not attend. | The woman cries and prays for her life in the church. |
| 3 | Harmony | 2010 | Kang, Dae-kyu | A woman who murdered her husband to prevent his domestic violence gave birth to a baby in prison. The woman had to send her baby away because of the regulation that babies born in prison must be placed for adoption at 18 months old. | The woman cries upon seeing the baby being taken into someone else’s hands. |

**Neutral Condition**

|  | Title | Year | Director | Background information | Content of video clip |
| --- | --- | --- | --- | --- | --- |
| 1 | Overman | 2015 | Seo, Eun-Young | A man volunteering at the library was surprised to see the enormous number of loans from a library patron around his age and asked the librarian who he was. The librarian told the man stories about various library patrons. | The man and the librarian talk about library patrons with many loans. |
| 2 | 10 Minutes | 2013 | Lee, Yong-seung | A man studied hard for the press exam every day to become a broadcasting producer, and he often works out in his spare time while studying. Today he went to his room right after dinner and studied for the exam. | The man leaves the house for a study break and exercises lightly. |
| 3 | Nobody's Daughter Haewon | 2013 | Hong, Sang-soo | A few days before leaving the country, a mother met her daughter, who was a college student living alone near campus. They walked around the neighborhood where the daughter lived. Finally, they went to the daughter’s campus and tried to look around. | The mother says that her legs are sore due to walking for a long time, so she and her daughter go to a cafe to drink hot tea. |
